# Storm effects on intertidal invertebrates: increased beta diversity of few individuals and species

**DOI:** 10.7717/peerj.3360

**Published:** 2017-05-23

**Authors:** Guilherme N. Corte, Thomas A. Schlacher, Helio H. Checon, Carlos A.M. Barboza, Eduardo Siegle, Ross A. Coleman, Antonia Cecília Z. Amaral

**Affiliations:** 1 Departamento de Biologia Animal, Universidade Estadual de Campinas, Campinas, São Paulo, Brasil; 2 School of Science and Engineering, University of the Sunshine Coast, Maroochydore DC, Queensland, Australia; 3 Núcleo em Ecologia e Desenvolvimento, Universidade Federal do Rio de Janeiro, Macaé, Rio de Janeiro, Brasil; 4 Instituto Oceanográfico, Universidade de São Paulo, São Paulo, São Paulo, Brasil; 5 School of Life and Environmental Science, University of Sydney, Sydney, New South Wales, Australia

**Keywords:** Benthos, Soft-bottom, Extreme events, Habitat heterogeneity, Araçá bay, Macrofauna, Climate change

## Abstract

Climate change is predicted to lead to more extreme weather events, including changes to storm frequency, intensity and location. Yet the ecological responses to storms are incompletely understood for sandy shorelines, the globe’s longest land-ocean interface. Here we document how storms of different magnitude impacted the invertebrate assemblages on a tidal flat in Brazil. We specifically tested the relationships between wave energy and spatial heterogeneity, both for habitat properties (habitat heterogeneity) and fauna (β-diversity), predicting that larger storms redistribute sediments and hence lead to spatially less variable faunal assemblages. The sediment matrix tended to become less heterogeneous across the flat after high-energy wave events, whereas β-diversity increased after storms. This higher β-diversity was primarily driven by species losses. Significantly fewer species at a significantly lower density occurred within days to weeks after storms. Negative density and biomass responses to storm events were most prominent in crustaceans. Invertebrate assemblages appeared to recover within a short time (weeks to months) after storms, highlighting that most species typical of sedimentary shorelines are, to some degree, resilient to short-term changes in wave energy. Given that storm frequency and intensity are predicted to change in the coming decades, identifying properties that determine resilience and recovery of ecosystems constitute a research priority for sedimentary shorelines and beyond.

## Introduction

Extreme weather events, including changes to storm frequency and intensity, are predicted to increase over the 21st century (IPCC, 2013; Lin & Emanuel, 2016; Walsh et al., 2016). These global changes to ecosystem physical and chemical conditions are having numerous and widespread biological impacts in the sea and on land (Weatherdon et al., 2016). In the global oceans, climate change is expected to substantially alter the provision of ecosystems services critical to humankind, such as coastal protection and capture fisheries (Gattuso et al., 2015), but many responses in marine ecoystems still remain incompletely understood (Hauser et al., 2016; Nagelkerken & Munday, 2016).

Storms may cause massive changes to coastal environments, particularly on sedimentary shorelines (Mateo & Garcia-Rubies, 2012), often causing the translocation of sediment from the beach and dunes, and the landwards movement of the coastline (Masselink et al., 2016). These large habitat changes are usually accompanied by impacts to faunal assemblages, best documented for benthic invertebrates, seagrass meadows, and algal communities (Lucrezi, Schlacher & Robinson, 2010; Jaramillo et al., 2012; Mateo & Garcia-Rubies, 2012).

The unpredictable nature of storms generally precludes the use of a rigorous experimental design to specifically test for storm effects, meaning that nearly all published ‘storm studies’ are largely opportunistic (Harris et al., 2011). In addition, often only a few or no data points are available immediately before a storm, post-storm sampling can be truncated, and for large storms it is challenging or impossible to find control areas that were not affected by the event (Posey et al., 1996); arguably, this makes attribution of ecological patterns to storm effects somewhat weak. An alternative is to make *a priori* predictive hypotheses based on knowledge of the biology of species and their likely response to large disturbance events in their habitat (Harris et al., 2011).

Here, we combine oceanographic, sedimentary and biological data to investigate how storms can affect the sedimentary habitat of a tidal flat in Southeast Brazil and the macrobenthic assemblages inhabiting it. Specifically, we tested four complementary, predictive hypotheses:

1. Higher wave energy during storms may translocate and disperse large sediment volumes (Masselink et al., 2016). We therefore predict that habitat heterogeneity (i.e., the spatial variation in seafloor properties amongst sampling sites) would be reduced after storms.
2. Habitat heterogeneity can be a major determinant for ecological assemblages, typically promoting beta diversity (i.e., variability in species composition among sampling units for a given area) (Anderson, Ellingsen & McArdle, 2006, Schlacher et al., 2007, McClain & Barry, 2010; Meager, Schlacher & Green, 2011). Therefore, we expect that storms lower beta diversity of the fauna.
3. Disturbance caused by storms has been reported to detrimentally affect populations of benthic species (Jaramillo, Croker & Hatfield, 1987; Mateo & Garcia-Rubies, 2012). Accordingly, we expect lower species richness, abundance, and biomass of invertebrates after storms.
4. Given that we expect lower β-diversity (prediction 2) and reduced number of species after storms (prediction 3), we predict that changes in β-diversity may be mainly attributable to species losses rather than species replacement.

### Material and Methods

### Study area

**Figure 1:**
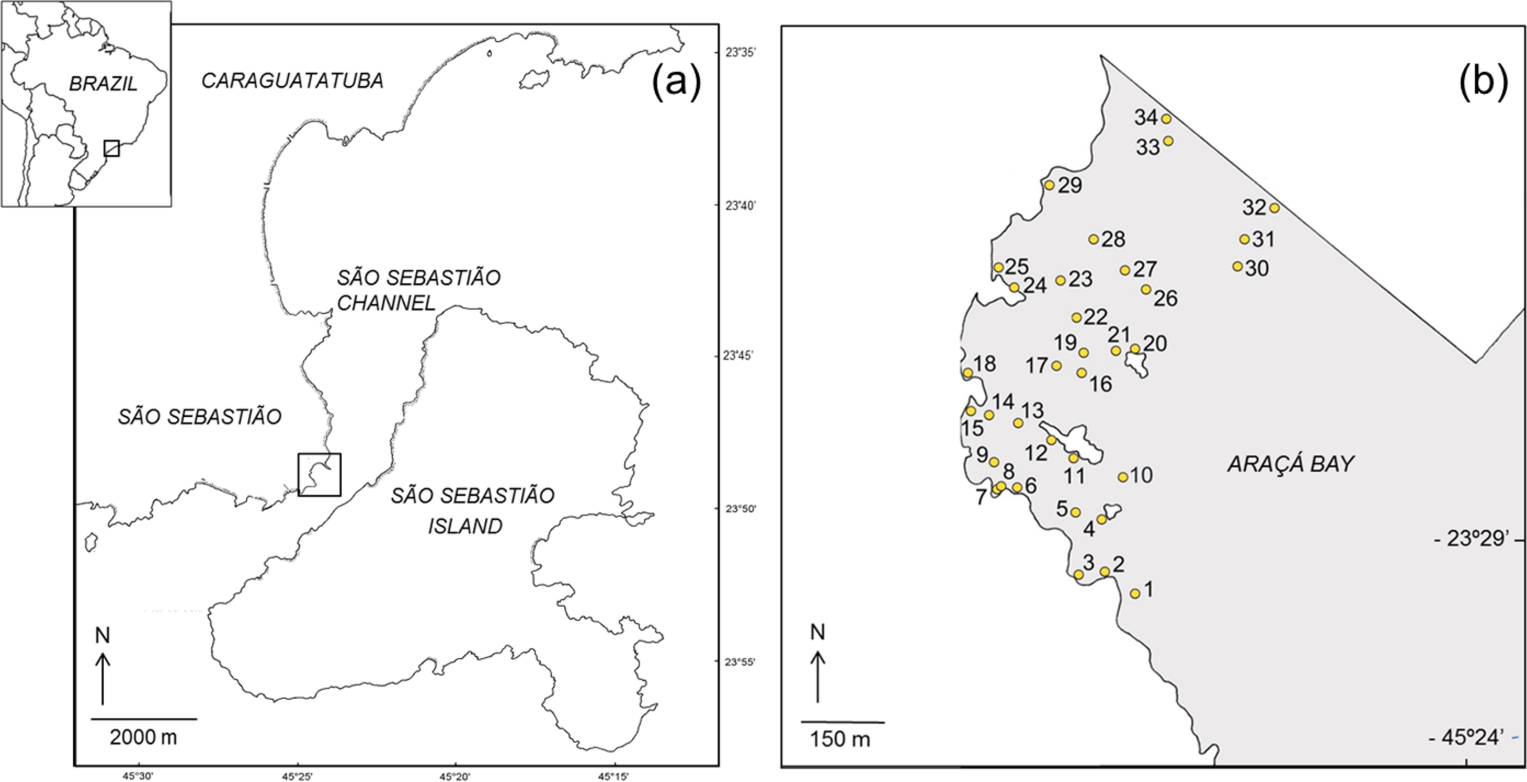
Map showing the location of the study area (A) and the sampling sites in the intertidal area of Araçá Bay (B).

This study was done on the intertidal flats of Araçá Bay (Brazil, 23°49′S, 45°24′W; Fig. 1), a sheltered and heterogeneous intertidal flat adjacent to the São Sebastião Channel, Southeast Brazil (Amaral et al., 2010). The area is relatively small (ca. 750 m wide and long) and protected from the prevailing swell by São Sebastião Island (Fig. 1). It is one of few tide-dominated environments along the southeastern coast of Brazil (Dottori, Siegle & Castro, 2015). Hydrographic properties of Araçá Bay are subject to physical forcing by frontal systems, when current speeds can increase eightfold (Fo, 1990). At the region, the highest storm waves are associated to cold fronts and reaching offshore significant wave heights of 6.4 m (Pianca, Mazzini & Siegle, 2010).

### Field sampling

**Figure 2:**
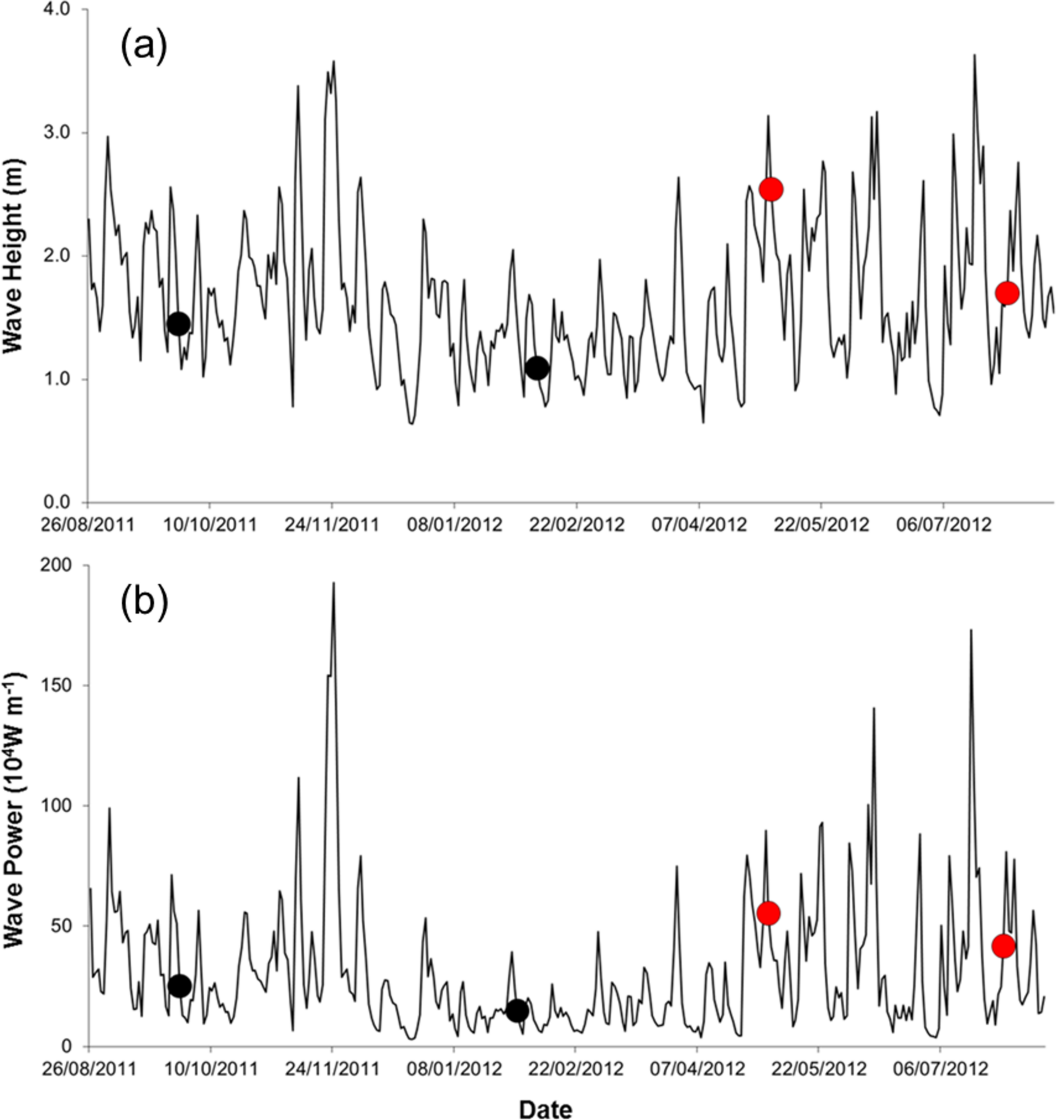
Wave height (A) and wave power (B) during the study period (sampling events are shown by dots. Red dots correspond to storm sampling events).

Field work was done during spring tides on four times, at ca. three month intervals, from September 2011 to July 2012 (25 September 2011, 5 February 2012, 7 May 2012, and 29 July 2012). Three storm events occurred during the study (22 November 2011, 06 May 2012, 18 July 2012; Fig. 2), all accompanied by torrential rain, strong winds, flooding, and building damages. We sampled on the first spring tide after the storms in May and July 2012 (one-day lag in May and 11 days in July).

During each sampling event, field work was done early in the morning of two consecutive days, collecting fauna from 34 sites. The sampling sites were positioned to encompass habitat diversity of the tidal flat (i.e., different sediment types over a range of depths), and to achieve a reasonable dispersion and spatial coverage (Fig. 1). The position of each sampling site was recorded with a GPS (Garmin eTrex Legend, *datum* WGS84) and the same locations (±1 m) were sampled during each of the four sampling events. Three faunal samples (corer: 20 cm inner diameter, 20 cm depth) and one sediment sample (corer: 3 cm inner diameter, 20 cm deep) were collected per site and event.

### Biological and environmental data

Fauna cores were washed on the same day of collection through a 0.3 mm mesh sieve, and the retained fauna was fixed in 70% ethanol. Sediment granulometric analysis was performed with standard dry sieving described by Suguio (1973). Sediment statistics were calculated with SysGran software (Camargo, 2006) using the parameters of Folk & Ward (1957). Organic matter content was determined by weight losses of dried samples (60 °C for 24 h) after incineration (550 °C for 6 h). Calcium carbonate content was determined by 10% HCl digestion.

Sediment temperature and interstitial water salinity were measured *in situ* with a digital thermometer and an analog refractometer (precision of 0.01 and 0.1 units, respectively). Wave height and period for the region were obtained for 24.5 S and 45.5 W from the global wave generation model WaveWatch III (NCEP/NOAA). Wave power (*P*_*w*_) was calculated as: *P*_*w*_ = *ρg*^2^*H*^2^*T*∕32*π*, where *ρ* is water density (1,027 kg/m^3^), g the acceleration due to gravity (9.81 m/s^2^), H the wave height (m), and T the wave period (s) (Herbich, 2000). We considered wave height and power for the three days before each sampling event. This time lag was found to show the strongest correlation between wave height/power and changes in macrobenthic species in the area (Turra et al., 2016).

All work was done in accordance with permit No. 19887-1 issued by the federal environmental agency, Ministério do Meio Ambiente, Instituto Chico Mendes de Conservação da Biodiversidade (ICM-Bio).

### Data analysis

We tested for differences in habitat heterogeneity and fauna β-diversity amongst times with permutational analysis of multivariate dispersion (PERMDISP, Anderson, 2006). In this analysis, higher multivariate dispersion is an indication of higher variability (i.e., higher habitat heterogeneity and β-diversity) among sampling sites (Anderson, 2006). For habitat heterogeneity, the test was based on Euclidean distances calculated from normalized sediment data. For fauna β-diversity, the test was based on Hellinger transformation (Legendre & De Cáceres, 2013) calculated from abundance data for the full suite of species. PERMDISP analysis was done using Primer 6 software (Clarke & Gorley, 2006). Ordination plots (nMDS) were computed with the vegan package in R (Oksanen et al., 2007) to illustrate differences in habitat heterogeneity and β-diversity (i.e., dispersion of sampling sites) between sampling events.

We tested for differences in species richness, abundance and biomass of invertebrate assemblages amongst times using general linear models with ‘Time’ as fixed factors. Models were adjusted using the negative binomial distributions for count data (species richness and abundance) and gamma distributions for continuous data (biomass). We used Tukey post-hoc tests to examine differences among sampling times using the MASS package in R (Ripley et al., 2013). This framework was used to investigate differences in the whole assemblage and also in the main groups of intertidal macrofauna (i.e., molluscs, polychaetes and crustaceans) separately.

We used the Similarity percentage analysis (SIMPER) based on Bray-Curtis distance to investigate the contribution of each individual species to the differences in species assemblages among sampling periods. Data was log(*x* + 1) transformed before analysis to reduce influence of abundant species. SIMPER was done in Primer 6 software (Clarke & Gorley, 2006).

We used the β-diversity partitioning framework of Podani & Schmera (2011) and Carvalho, Cardoso & Gomes (2012) to investigate compositional changes of macrobenthos (i.e., β-diversity) over time. This framework calculates compositional differences among communities (β_total_) and partitions it into βdiversity attributed to species replacement (β_repl_) and βdiversity attributed to species loss or gain (β_rich_). This analysis was done with the R package BAT (Cardoso, Rigal & Carvalho, 2015).

## Results

Seawater temperature varied seasonally, whereas salinity and organic matter content of the sediment changed relatively little over time (Table 1). The silt, clay, and fine sand fraction of the sediment increased between Sep. 2011 and July 2012 (Table 1). Waves were higher and more powerful before samplings in May and July 2012 (Table 1, Fig. 2).

**Table 1:** Environmental parameters recorded.

	September 2011	February 2012	May 2012	July 2012
	mean (se)	mean (se)	mean (se)	mean (se)
Temperature (°C)	21.9 (0.2)	27.4 (0.2)	25.0 (0.2)	20.4 (0.1)
Salinity	32.3 (0.3)	31.7 (0.9)	30.6 (0.7)	29.9 (0.6)
Mean grain size (*ϕ*)	2.5 (0.7)	2.7 (0.7)	2.7 (0.5)	2.8 (0.64)
Silt and clay (%)	4.2 (0.6)	4.7 (0.6)	4.8 (0.6)	5.7 (0.9)
Fine sand (%)	68.4 (3.2)	73.5 (3.2)	74.1 (3.8)	74.7 (3.1)
Coarse sand (%)	10.7 (1.6)	9.5 (1.7)	7.9 (1.3)	7.3 (1.6)
Pebbles (%)	6.2 (1.4)	3.7 (1.1)	3.2 (0.9)	3.2 (1.0)
Organic matter (%)	1.6 (0.1)	1.7 (0.2)	1.7 (0.2)	1.9 (0.2)
CaCO_3_ (%)	4.9 (0.4)	4.4 (0.4)	3.8 (0.5)	3.5 (0.3)
Height of waves (m)	1.5 (0.06)	1.6 (0.04)	2.1 (0.11)	1.7 (0.04)
Power of waves (10^4^ W/s)	20.1 (1.7)	18.1 (7.3)	42.8 (5.3)	30.4 (3.4)

We recorded 126 species from 33,320 individuals during the study (Data S1). Polychaetes, molluscs and crustaceans made up 94% of species (polychaetes: 67 species; molluscs: 34 species; crustaceans: 18 species). Crustaceans were the most abundant group, comprising 56.5% of all individuals, mainly because of the high number of the tanaidacean *Monokalliapseudes schubartti* (Mañé-Garzón, 1949); polychaetes made up 39.6%, and molluscs 3.7% of catches (Data S1).

### H1: lower habitat heterogeneity after storms

**Figure 3:**
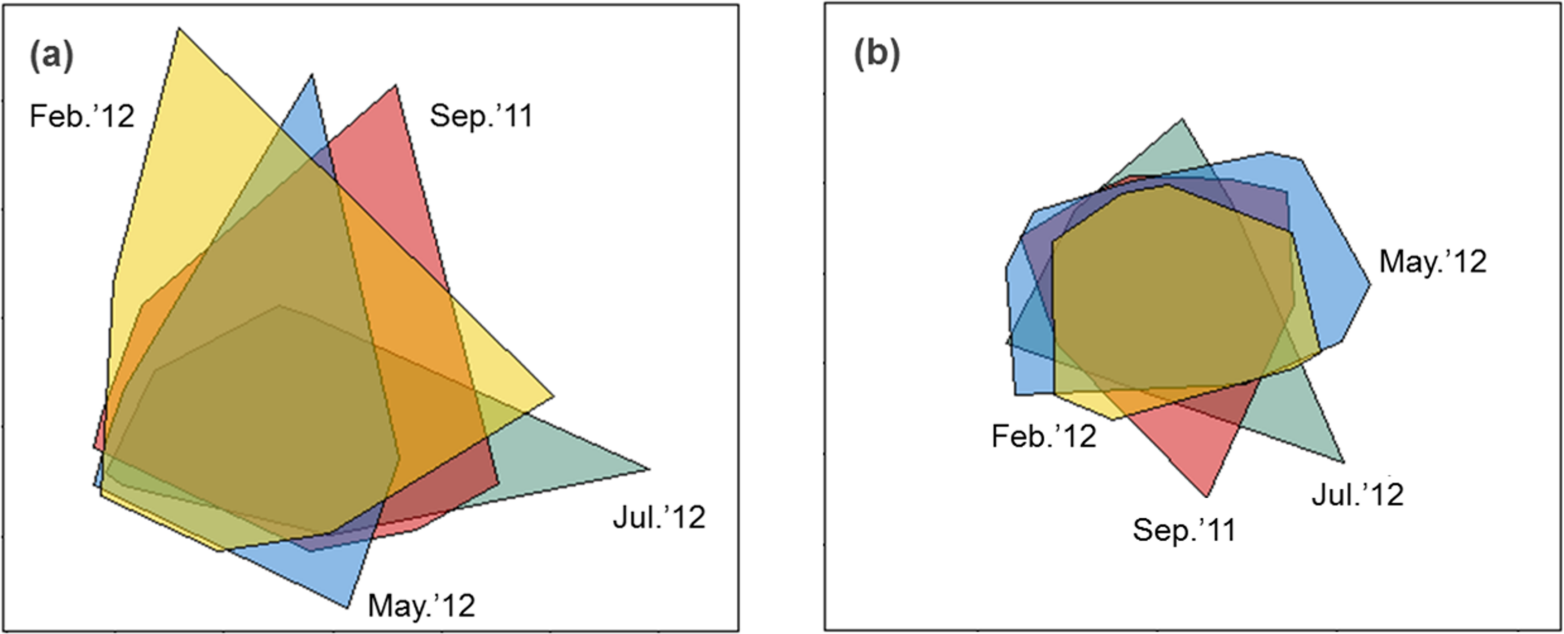
nMDS biplot of Euclidean distance based normalized environmental data (A) and nMDS biplot of Hellinger disimilarity matrix based on macrobenthic abundance data (B). Samples from September 2011 are located inside the polygon plotted in red, from February 2012 in yellow, from May 2012 in blue, and from July 2012 in green. Stress for these ordination are 0.10 (A) and 0.19 (B).

**Figure 4:**
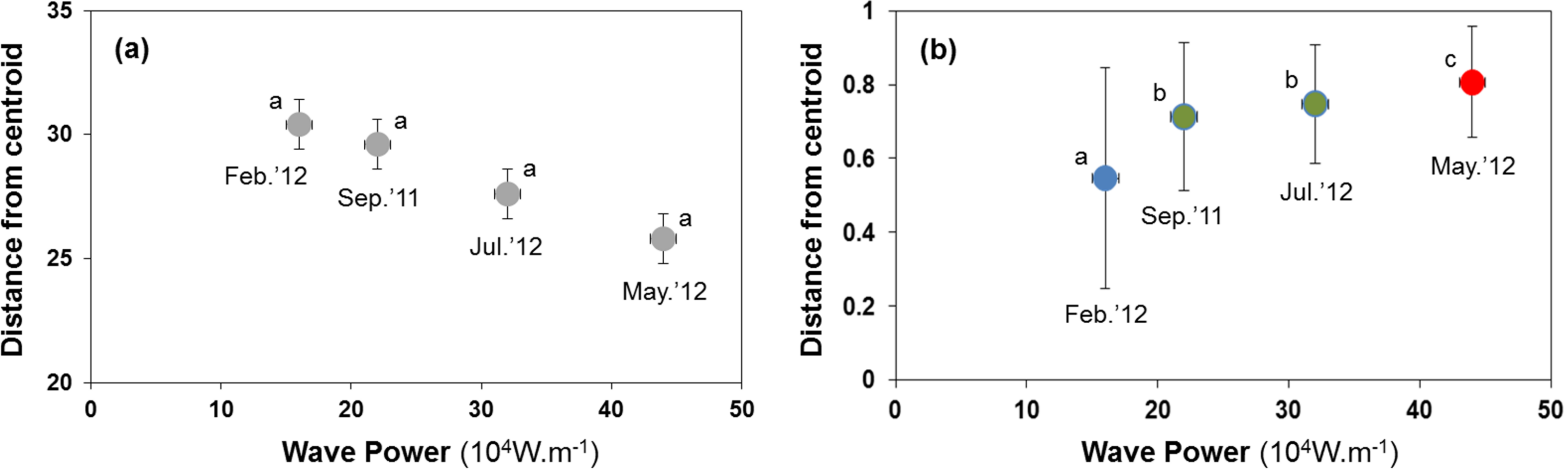
Habitat heterogeneity (A) and β-diversity of macrobenthic assemblages (B) during four sampling events associated with significant variation in wave energy preceding each event. Measure of habitat heterogeneity and β-diversity is the distance from centroids across all sites at a time. Letters and colours denote different groups (*P* < 0.05) in permutational analysis of multivariate dispersion (PERMDISP). Error bars denote standard errors.

Sediment properties were spatially more homogeneous after periods of higher wave power (Fig. 3A), but differences between sampling times were not significant (Fig. 4A; PERMDISP *P* = 0.586).

### H2: B-diversity declines after storms due to more homogenous sediment matrix

Macrobenthic assemblages showed a significantly higher βdiversity following periods of higher wave power (Figs. 3B and 4B; PERMDISP *P* = 0.001). Species that contributed most to differences in macrobenthic assemblages among sampling periods are listed in Tables 2 and 3. Storm effects appear to be driven mainly by large declines in the abundance of the tanaid *Monokalliapseudes schubartti*, whereas polychaetes (i.e., *Capitella* sp.C, *Heteromastus filiformis, Armandia hossfeldi*) tended to increase in abundance following storm events (Tables 2 and 3).

**Table 2:** Similarity percentage (SIMPER) analysis showing the contribution (%) of the five most important species to differences in species assemblages among sampling periods.

	*Monokalliapseudes Schubarti*	*Capitella* sp. C	*Heteromastus filiformis*	*Scoloplos* sp1	*Armandia hossfeldi*	Mean dissimilarity
Sep. vs. Feb.	10.98	4.96	n/a	5.34	n/a	68.1
Sep. vs. May	11.14	6.55	n/a	5.46	n/a	74.9
Sep. vs. July	9.65	6.85	5.51	5.27	6.47	69.3
Feb. vs. May	16.03	8.17	5.04	5.44	n/a	75.3
Feb. vs. July	12.75	7.9	5.9	5.37	7.24	71.6
May vs. July	8.29	8.97	6.63	6.22	8.04	69.9

**Table 3:** Temporal variation in the density (ind.m^−2^) of species that accounted for most of the assemblage-wide differences in macrobenthic assemblages among sampling events (cf. Table 2).

	September 2011	February 2012	May 2012	July 2012
	mean (se)	mean (se)	mean (se)	mean (se)
*Monokalliapseudes schubartii*	2,151 (635)	3,264 (571)	1,776 (79)	538 (276)
*Capitella* sp.C	114 (68)	521 (257)	667 (428)	768 (336)
*Heteromastus filiformis*	23 (9)	32 (11)	47 (12)	133 (36)
*Scoloplos* sp1	112 (30)	63 (14)	35 (10)	87 (19)
*Armandia hossfeldi*	50 (25)	7 (4)	47 (19)	224 (74)

### H3: storm disturbance results in lower abundance, biomass, and species richness

**Figure 5:**
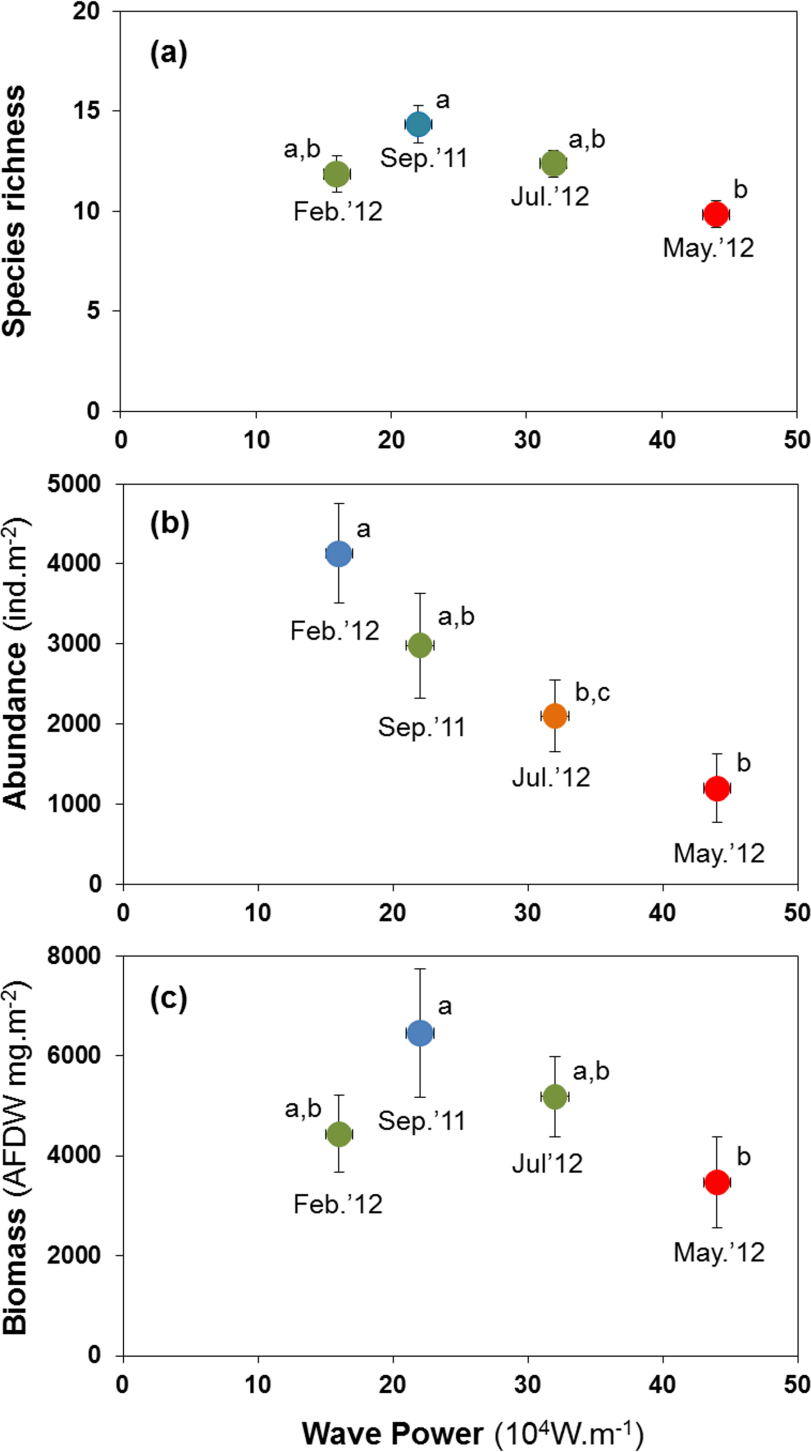
Variation in the mean number of species per site (A), mean abundance (B) and mean biomass (C) of macrobenthic assemblages at four sampling events associated with significant variation in wave energy preceding each event. Letters and colours denote different groups in generalized linear models (*P* < 0.05). Error bars denote standard errors.

Abundance, species richness and biomass were significantly lower in samples taken shortly after high-energy wave events (Fig. 5). The mean number of species per site was lowest at 9.82 species after the strongest wave event, compared with 11.82 to 14.35 species at other times (Fig. 5A). Abundance peaked at 4,126 ind.m^−2^ in Feb. 2012, declining to 1,195 ind.m^−2^ after the storm in May 2012 (Fig. 5B). Biomass declined from 6.5 to 3.5 gAFDW.m^−2^ between September 2011 and May 2012 (Fig. 5C).

**Figure 6:**
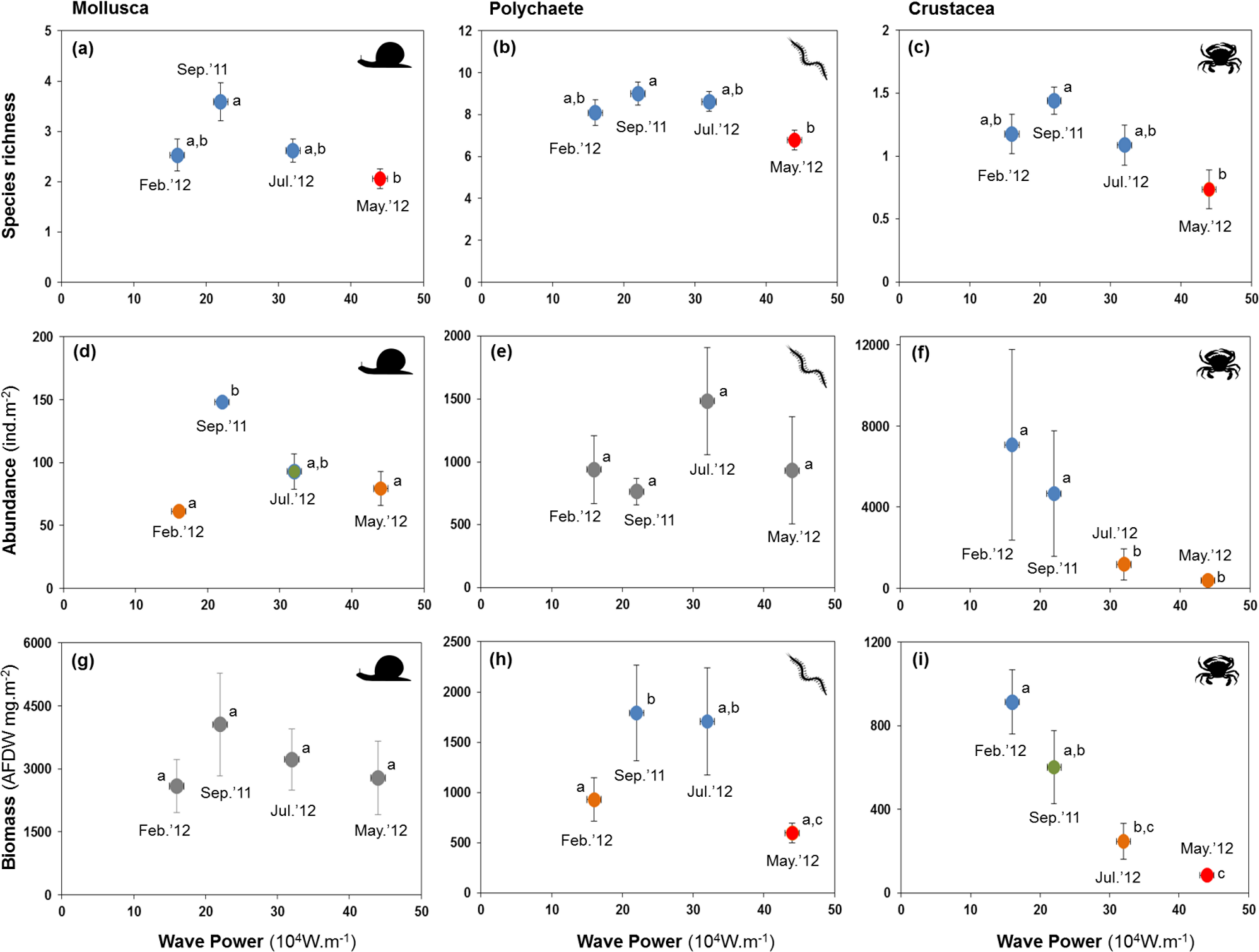
Variation in the mean number of species, abundance, and biomass of molluscs (A, D and G), polychaetes (B, E and H), and crustaceans (C, F and I) at four sampling events associated with significant variation in wave energy preceding each event. Letters and colours denote different groups in generalized linear models (*P* < 0.05). Error bars denote standard errors.

All major groups of intertidal macrofauna (i.e., molluscs, polychaetes, crustaceans) were affected by storms (Fig. 6). The mean number of species per site of all groups was significantly lower after the strongest wave event (*P* < 0.05) (Figs. 6A–6C). Temporal patterns of changes in abundance and biomass did, however, differ between groups. Crustaceans showed the most pronounced density (Fig. 6F) and biomass (Fig. 6I) response, declining strongly after storms. Molluscs showed a broadly similar density pattern to crustaceans, albeit being less pronounced (Figs. 6D and 6G), whereas the abundance of polychaetes tended to increase following periods of higher wave energy (Figs. 6E and 6H).

### H4: species losses drive most of the change in β-diversity

Declines in species numbers accounted for most of temporal β-diversity in the macrobenthos, and its contribution was higher shortly after storms (Table 4). By contrast, species replacement was less important.

## Discussion

Significant changes in macrobenthic species richness, abundance and biomass in a tropical tidal flat were associated with storms. This resulted in significant changes to fauna β-diversity over time that was mainly attributable to species losses, but not strongly linked to variation in habitat heterogeneity.

Previous studies about the influence of storms on coastal soft-sediment ecosystems have shown that storms may have stronger impacts on environmental features than on the fauna (e.g., Saloman & Naughton, 1984; Cochôa, Lorenzi & Borzone, 2006; Alves & Pezzuto, 2009; Harris et al., 2011), and that offshore sediment transport is the dominant geo-morphological response of sedimentary shores to increased wave energy (Masselink et al., 2016). These studies were, however, mostly done on exposed ocean beaches, habitats with fewer species that are well adapted to high-energy conditions (Brown, 1996; Schlacher et al., 2008). By contrast, our results showed that under more sheltered conditions, storm impacts were more evident for the fauna than for the environment.

**Table 4:** B-diversity and β-diversity partitioning among sampling periods. Higher values of β-diversity denote greater differences in the composition of species and number of individuals between two consecutive sampling times. Percentages indicate the amount of variation between periods attributable to species losses or species replacement.

	Total β diversity (β_total_)	Species replacement/ substitution (β_repl_)	Species loss/gain richness differences (β_rich_)
Sep. vs Feb.	0.45	44.4%	56.4%
Feb. vs May	0.79	16.4 %	83.6 %
May vs July	0.47	11.7 %	89.3 %
**mean**	0.57	24.2 %	76.4 %

The observed decrease in the number of species, individuals and biomass of macrobenthic assemblages may have been caused by accretion and redistribution of sediments, burying fauna at some site and winnowing them from others. Waves and currents may suspend fine-grained sediments (Lohrer, Hewitt & Thrush, 2006), and alongshore sediment distribution within the same system or accretion of sediment in washover deposit can occur after storms (Masselink et al., 2016). Moreover, Alcántara-Carrió et al. (2017) showed that the seaward transport of terrigenous sediment after intense rains in combination with resuspension of sediments by storm waves and transport by wind-driven currents alter the sedimentary features in the São Sebastião Channel. These hypothesized mechanisms of fauna change are functionally supported by studies showing significant changes to the macrobenthos following sediment deposition and substantial alterations in hydrodynamic regimes (Jaramillo et al., 2012; Cummings et al., 2003; Rodil et al., 2011; Schlacher et al., 2012).

Whilst storms were followed by decreases in species richness in all major groups of the macrobenthos, changes in species richness were more pronounced in crustaceans, which also declined strongly in abundance and biomass. This was mainly a consequence of massive (−95%) declines of the tanaid *Monokalliapseudes schubarti. M. schubarti* is small (ca. 5 mm) and builds tubes that rarely extend for more than 5 cm into the sediment, possibly making it more susceptible to sediment erosion (Nucci, Turra & Morgado, 2001). In fact, morphological traits of benthic invertebrates have been suggested to modulate storms impacts (Mateo & Garcia-Rubies, 2012), with small-bodied individuals and those with low mobility thought to be more susceptible to storms (Negrello Filho & Lana, 2013; Urabe et al., 2013).

Fewer species of polychaetes were recorded at lower biomass after storm events. Some species, mostly small and tubiculous forms such as *Isolda pulchella* (Müller in Grube, 1858), were less numerous after storms. By contrast, opportunistic polychaete species (e.g., *Capitella* spp, *Heteromastus filiformis* (Pearson & Rosenberg, 1978)) increased in abundance after storms. We did not reccord significant changes in the biomass of molluscs, possibly a consequence of heavier, shelled forms of the macrobenthos being less likely to be displaced by turbublent currents associated with storms.

The relationship between wave power and changes in macrobenthic fauna metrics approximated in several cases a bell-shaped curve, suggesting a resemblance with the “intermediate disturbance hypothesis” (IDH, Connell, 1978); a core prediction of IDH is that at high disturbance levels species intolerant of the disturbance become locally extirpated whereas more intense competition limits species numbers at low disturbance intensity and frequency. Consequently, the highest number of species is expected to occur at intermediate levels of disturbance (but see Fox (2013), Sheil & Burslem (2013), and Huston (2014) for discussions on the validity of the IDH). Arguably, disturbance by wave energy at intermediate levels may have enhanced species richness and productivity at sedimentary coastal ecosystem by reducing competition. Nevertheless, as pointed out by Huston (2014), the causes of high diversity go beyond the simple effects of disturbances slowing the process of competitive exclusion and must include multiple ecological and evolutionary processes. In sedimentary shorelines, especially in sheltered environments, intermediate disturbance caused by waves is expected to increase water circulation processes and may also enhance biodiversity and productivity of macrobenthic assemblages by increasing the amount of food available in the water column, and/or reducing the concentration of nutrients in the sediment, resulting in lower eutrophication processes (Cloern, 2001; Corte, Coleman & Amaral, 2017).

Storm effects appeared to be influenced by the timing of a storm in relation to the tidal regime. Masselink et al. (2016) found that storms impacts on the south-west coast of England were highest when the peak storm waves coincided with spring high tides. In our study, this was likely the case in May 2012 when the height of the storm passed during a spring tide. The observed effects of storms on the macrobenthic fauna of Araçá Bay also appeared to be stronger during a short time after storms. We found that differences in environmental and biotic characteristics were most pronounced in May 2012, when samples were taken one day after the storm had passed.

Most species typical of sedimentary shorelines are, to some degree, adapted to high-energy conditions and hence may recover relatively quickly (e.g., within days to weeks) from most storm events (Harris et al., 2011; Schlacher & Thompson, 2013; Machado et al., 2016). For example, in a subtropical coastal soft-sediment ecosystem in South Brazil, Gallucci & Netto (2004) found that abundance and number of species of macrobenthic organisms declined during the passage of a cold front, but all values were back to pre-frontal conditions within a day. Similarly, Machado et al. (2016) found recovery of macrobenthic assemblages inhabiting tropical ocean exposed beaches within seven weeks of a storm. It is important to emphasize, however, that recovery depends on the magnitude, spatial scale and return frequency of the disturbance events in soft-sediment environments and other marine systems (Lucrezi, Schlacher & Robinson, 2010; Urabe et al., 2013; McClain & Schlacher, 2015; Schlacher et al., 2015). The most powerful storms may cause ecological changes that require years to recover and may compromise the spatial and trophic structure of the ecosystems (Jaramillo, Croker & Hatfield, 1987; Mateo & Garcia-Rubies, 2012).

## Conclusion

Here we show that storms can cause significant changes to macrobenthic assemblages inhabiting a tidal flat. Decreases in species richness, abundance, and biomass of invertebrate assemblages were related to increases in wave power. Species losses drove changes towards higher β-diversity, but the fauna appeared to recover within a few weeks. Changes in habitat features were comparatively smaller. Given that storm activity, location and intensity are predicted to change over the coming decades in a warming world (Lin & Emanuel, 2016; Walsh et al., 2016), ecological changes attributed to altered storm properties are likely. Unfortunately, the functional consequences of altered storm regimes for coastal ecosystems are largely unknown, including the continued provision of ecosystem services such as coastal protection and capture fisheries. Thus, future work shall prioritise investigations of how ecological processes in coastal ecosystems respond to extreme events and which features may determine their resilience and recovery.

##  Supplemental Information

10.7717/peerj.3360/supp-1 Data S1 Supplementary data

## Additional Information and Declarations

### Field Study Permissions

The following information was supplied relating to field study approvals (i.e., approving body and any reference numbers): All activities complied with the license from the appropriate federal environmental agency (Ministério do Meio Ambiente (MMA)—Instituto Chico Mendes de Conservação da Biodiversidade (ICMBio) No. 19887-1; acronyms for, in English: Ministry of the Environment—Chico Mendes Biodiversity Conservation Institute).

### Data Availability

The following information was supplied regarding data availability: The raw data has been supplied as Data S1.
